# Shaping immunity: the influence of the maternal gut bacteria on fetal immune development

**DOI:** 10.1007/s00281-025-01039-8

**Published:** 2025-02-01

**Authors:** Marijke M. Faas, Alexandra M. Smink

**Affiliations:** Department of Pathology and Medical Biology, University Medical Center Groningen and University of Groningen, Hanzeplein 1, Groningen, 9713 GZ The Netherlands

**Keywords:** Fetus, Immune response, Maternal gut bacteria, Pregnancy, Prebiotics and probiotics

## Abstract

The development of the fetal immune response is a highly complex process. In the present review, we describe the development of the fetal immune response and the role of the maternal gut bacteria in this process. In contrast to the previous belief that the fetal immune response is inert, it is now thought that the fetal immune response is uniquely tolerant to maternal and allo-antigens, but able to respond to infectious agents, such as bacteria. This is accomplished by the development of T cells toward regulatory T cells rather than toward effector T cells, but also by the presence of functional innate immune cells, such as monocytes and NK cells. Moreover, in fetuses there is different programming of CD8 + T cells and memory T cells toward innate immune cells rather than to adaptive immune cells. The maternal gut bacteria are important in shaping the fetal immune response by producing bacterial products and metabolites that pass the placenta into the fetus and influence development of the fetal immune response. Insight into how and when these products affect the fetal immune response may open new treatment options with pre- or probiotics to affect the maternal gut bacteria and therewith the fetal immune response.

## Introduction

The fetal immune response is a complex and highly regulated process that plays an important role in fetal development. It maintains tolerance to self-antigens as well as to maternal and allo-antigens and, at the same time, protects the fetus from pathogens. Neonates show an increased risk for infections and infectious diseases are the most common cause of neonatal death [1]. This was previously thought to result from an immature immune system at birth: it was believed that although immune cells, such as T cells, were present at birth, they were not able to respond to activation stimuli with the production of cytokines since neonatal T cells did not produce Th1 cytokines, such as interleukin (IL)−2 or interferon γ (IFNγ) [2–4]. Furthermore, it was believed that there was no need for an immune response as the fetus was sterile.

In the late 90s, studies found that cord blood T cells, representative of fetal T cells, could produce Th2 cytokines, such as IL-4 and IL-5, upon stimulation [5, 6]. This changed the paradigm from fetal T cells being immature to fetal T cells being different from adult cells: fetal T cells showed immune deviation toward development of Th2 cells. This theory also explains why neonates are more susceptible to infections, especially to intracellular infections, since Th1 immune responses are important for clearing such infections [7]. This theory has also been the basis for the hygiene hypothesis, which suggests that lack of contact with infectious agents, such as bacteria and viruses, in early childhood, increases the susceptibility to allergic diseases by a lack of Th1 development [8].

Most recent evidence suggests that fetal T cells are a special subset of T cells, with unique functions important for fetal life. The fetal adaptive immune response is tolerant against self, but also toward maternal antigens and does not induce a proinflammatory response toward these antigens [9]. This tolerant immune system is important for fetal development since a strong fetal immune response against self or maternal antigens could induce fetal rejection. However, at the same time, the innate fetal immune response, when needed, can respond to infections and microbes [10–12]. This latter innate immune response development is also important since after birth the fetus must immediately be able to respond to environmental and microbial exposures. The transition of the adaptive immune response from tolerance to protection after birth takes time and makes the neonate sensitive to infections.

The development of an innate immune system in the fetus suggests that the fetus comes into contact with antigens and microbes. Thus, the fetus may not be sterile, but exposed to antigens. Various studies have shown that antigens derived from maternal cells trafficking to the fetus, from ingested amniotic fluid, food antigens, or bacteria or their products [13–16] may be important for properly developing the fetal immune response. As a consequence of these findings, it may be hypothesized that a lack of these antigens or the presence of abnormal antigens may result in aberrant development of the fetal immune system and accordingly development of an aberrant adult immune system. An aberrant immune system may result in the immune-mediated diseases, such as allergic diseases, in the child or adult.

Therefore, it is important to gain insight into how the fetal immune system develops, how appropriate development is induced, and if and which antigens participate in proper immune development in the fetus. In this review, we will start by introducing the immune system, followed by describing the development of fetal immune cells and the characteristics of the fetal immune responses. Subsequently, we will provide an overview of the role of the maternal gut bacteria and its products in shaping the fetal immune response. Therefore we will first describe the changes that occur in the maternal gut microbiome during pregnancy, followed by an evaluation of the presence of bacteria, their products or metabolites in the fetus. We will describe how the microbial products and metabolites influence (fetal) immune cells. Finally, we will examine the impact of maternal gut dysbiosis on fetal immune responses as well as the effects of maternal treatments with prebiotics and probiotics on the fetal immune system.

## The immune response

The immune system consists of an innate immune system and an adaptive immune system. One of the major roles of the innate immune system is to protect our body against pathogens. Innate immune cells can recognize pathogens and their products directly and respond to them. It is non-specific, important in the early immune response, and its main purpose is to prevent the spreading of foreign pathogens throughout the body [17]. The cells of the innate immune system are myeloid cells, including neutrophils and monocytes [17], innate lymphoid cells (ILCs) [18], and antigen-presenting cells (APCs), such as macrophages and dendritic cells. APCs are important in activating the adaptive immune response [17]. These APCs detect and kill pathogens by phagocytosis, followed by presentation of the pathogen’s antigen to T and B cells of the adaptive immune system [17].

The adaptive immune system consists of T and B lymphocytes. T cells are responsible for the cell-mediated immune response, while B cells conduct the humoral immune response. The adaptive immune response can recognize foreign or infected cells and is able to discriminate between self and non-self (i.e., allo-antigens) by using MHC molecules present on each mammalian cell. Conventional T cells (alpha/beta (αβ) T cells) can be divided into 2 groups, the CD8 + and CD4 + T cells. The CD8 + T cells, also known as cytotoxic T cells, are cytotoxic and are capable of inducing apoptosis in infected or stressed cells [17]. The CD4 + cells, also known as T helper cells, ‘help’ in regulating the immune response by producing cytokines and chemokines to guide and activate other immune cells [17]. CD4 + T cells can be subdivided into subgroups, such as Th1, Th2, and Th17, which help in cell-mediated immune responses, in humoral and allergic responses, and in response to extracellular bacteria, respectively [19]. Another subgroup are the regulatory T cells (Tregs), which help with controlling the immune response to avoid major damage to the surrounding tissues or suppress reactions against self-antigens [19]. Gamma/delta (γδ) T cells are a specific set of T cells with innate immunity-like characteristics, which recognize a broad spectrum of molecules in an MHC-independent manner and are able to respond to microbial stimuli [20]. The other lymphocyte subset within the adaptive immune response are B lymphocytes, which produce antibodies that bind to pathogens and thereby trigger other immune cells to destroy the pathogen [17]. There are two populations of B cells, B1 and B2 cells. B1 cells are found in peritoneal and pleural cavities and around the lungs and intestines [21]. B1 cells produce natural antibodies and are innate-like cells [21]. B2 cells are circulating B cells which are short-lived and can be activated in secondary lymphoid organs to collaborate with T cells to produce specific antibodies [21]. Activated naïve T or B cells develop into effector cells, which eliminate the pathogen. After elimination of the pathogen, most effector T and B cells die, however, some effector cells develop into memory cells, which are long-lived and able to quickly respond upon reoccurrence of the infection [17].

## Development of fetal immune cells

**Fig. 1:**
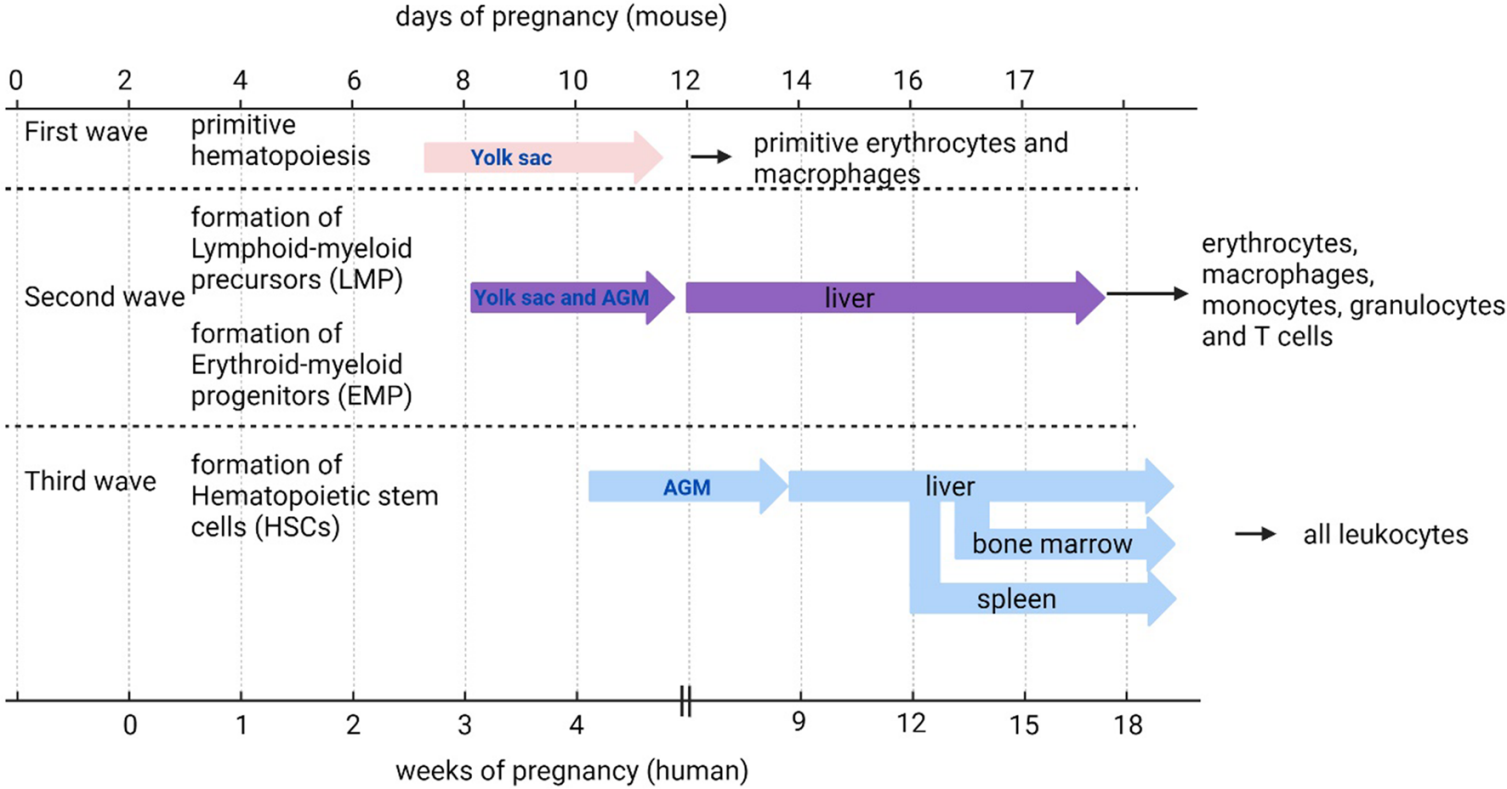
Development of fetal immune cell precursors in human and mouse pregnancy. Fetal immune cell precursors arise from the yolk sac or the aorta gonad mesonephros (AGM) region in three different waves. LMPs and EMPs as well as HSCs migrate to the liver before they start proliferating and developing into immune cells. HSCs migrate to the bone marrow and spleen later in pregnancy. (Created with Biorender)

Research into the development of human fetal immune cells is obviously hampered by ethical and safety concerns. However, the mammalian production of blood cells, i.e. hematopoiesis, is a highly conserved process [22] and much of our knowledge is derived from mouse embryos. Our description of hematopoiesis is therefore often based on mouse data, with complementary human data wherever possible (see also Fig. 1). Hematopoiesis occurs during fetal development at various sites: the yolk sac, the aorta gonad mesonephros (AGM) region, the liver, the bone marrow, and the placenta [23]. Fetal hematopoiesis is important for the production of red blood cells and leukocytes in the fetus, but also to establish a pool of hematopoietic stem cells (HSCs) for adult life. In mice, at embryonic day 7.5–8 (humans, day 16–19 of pregnancy), primitive hematopoiesis starts in the yolk sac [23–25]. During this process, primitive erythrocytes are formed, although macrophages and megakaryocytic lineages are also obtained [25–28]. This first primitive wave of hematopoiesis is important for immediate fetal development since primitive erythrocytes are important for tissue oxygenation and loss of these primitive erythrocyte precursors results in embryonic death [29].

The primitive hematopoiesis in the yolk sac is followed by a second wave of hematopoiesis in the yolk sac giving rise to more definite erythrocytes as well as myeloid cells, such as macrophages. These cells are called erythro-myeloid progenitors (EMPs) [28, 30]. In this second wave also lymphoid-myeloid precursors (LMPs) are formed [31], which produce lymphocytes and myeloid cells. Around the same time, the AGM region of the embryo contains HSCs that can differentiate into all blood cell types [25, 32]. EMPs from the yolk sac as well as the HSCs from the embryo migrate to the fetal liver [33, 34], which is the largest site of hematopoiesis in the fetus. In the fetal liver, these progenitors extensively proliferate [34] as it provides the optimal environment for expansion of these cells [35]. The most important function of these cells is to support embryonic growth and development and perform hematopoiesis in the fetal liver. HSCs finally migrate to the spleen and bone marrow around embryonic days 16–17 in mice [36, 37], which is also the major site of hematopoiesis in adults [38]. In humans, functional HSCs colonize the bone marrow around 12 weeks of gestation [39] (Fig. 1).

### Development of T cells

Thymic development begins around week 4–5 of pregnancy and αβ T cell cells (giving rise to the CD4 + and CD8 + T cells) and γδ T cells start to migrate from the fetal liver toward the thymus at 8 weeks of gestation [40, 41]. These T cells will mature and after the selection process in the thymus will join the peripheral blood and lymph system by week 12–14 of gestation [40, 41]. Mature CD4 + and CD8 + T cells can be detected in the fetus after 20 weeks of gestation [42, 43]. The majority of T cells in the fetus are naïve T cells, however, during the second trimester, Treg cells are also present and are enriched in the fetal circulation as compared with the adult circulation [42, 44, 45]. Interestingly, fetuses also contain memory T cells. Few memory T cells can be found in the cord blood (less than 5%) [46], while fetal intestines contain larger amounts of memory T cells [47–49]. The other set of T cells, γδ T cells, were enriched in fetal blood before 30 weeks of pregnancy as compared with adult blood [50], however, their percentage decreases during the course of pregnancy [50]. These γδ T cells express a TCR receptor containing the γ-chain variable region 9 and δ-chain variable region 2 (Vδ2+) [50]. This receptor recognizes microbe and bacterial-derived phospho-antigens [51, 52] and they showed an effector potential in the fetus [50, 53].

In human cord blood, the total number of T cells is higher when compared to adult blood [54] (Table 1). The T cell population of cord blood consists of αβ T cells and γδ T cells. αβ T cells consist of CD4 + T helper cells and CD8 + cytotoxic T cells, with a relatively high level of naïve T cells [55]. Cord blood CD4 + T cells are polarized toward the production of Th2 cytokines, while the production of Th1 cytokines is decreased [56, 57]. The cord blood contains only low levels of Th17 cells [58]. Treg cells, on the other hand, are more frequently found in cord blood as compared with adult blood [42]. Cord blood also contains a small number of CD4 + memory cells (1–3% of the CD4 + cell population) [46, 59]. These cells could be activated to produce Th1 and Th2 cytokines [46, 59]. Cord blood CD4 + T cells are also enriched in recent thymic emigrants (RTEs), which are T cells that have recently completed intrathymic development and regressed into the circulation [60]. These RTEs are immature CD4 + T cells and require about 3 weeks in the circulation to become mature T cells [61]. The number and function of cord blood CD8 + cells are decreased as compared to adult blood [62]. They have a different gene profile compared with adult CD8 + T cells, are less cytotoxic, and biased toward an innate immune response [63]. However, they can mount adult-like responses to viral infections [64]. Cord blood γδ T cells are present in low frequency, < 1%, have a naïve phenotype and are mainly Vδ2+ [65].

**Table 1:** Leukocyte subsets in human cord blood

leukocytes	subsets	numbers compared with adult blood	comments
T cells		↑	T cells are mostly naive
	αβ T cells CD4+	↑	CD4 + are mostly naive
	Th1	↓	
	Th2	↑	
	Th17	↓	Very little Th17 cells
	Treg	↑	
	RTE	↑	
	memory CD4+	↓	< 1%
	αβ T cells CD8+	↑	mostly naive and biased toward innate immune response
	memory CD8+	↓	< 1%
	γδ T cells	=	< 1%; Mostly Vδ2+
B cells		↑	B cells are mostly naive
	B1	↑	
	B2	↓	
	memory B cells	↓	Very little memory cells
ILCs		↑	
	NK cells	↑	No differences in percentage of the NK cell subsets
myeloid cells		↑	
	dendritic cells	↓	
	monocytes	↑	No differences in percentage of the monocyte subsets
	granulocytes	↑	

*Treg*; regulatory T cells, *RTE;* Recent Thymic Immigrants, *ILCs;* Innate Lymphoid Cells, *NK cells;* Natural killer cells

### Development of B cells

In the fetus, both B1 and B2 cell populations are present. However, there are mainly B1 cells, whereas in adults the largest part of the B cell population is B2 [21, 66, 67]. In mice, the development of B1 progenitors starts in the yolk sac and the aorta [68] and is found later in gestation in the bone marrow and fetal liver [69]. Although the exact timing of B1 cell development in humans has to be established, in the human fetus, B cells can be detected in the fetal liver at around 8 weeks of gestation [70], in the circulation at week 12 of gestation [71], and in the spleen at 13–23 weeks of gestation [70, 72].

The total number of B cells in human cord blood is higher as compared with adult blood [54] (Table 1). B cells in cord blood are mainly naïve and only have a partially developed expression of immunoglobulins on their surface [73]. Similar to the fetus, cord blood contains a high percentage of B1 cells (50%) [73]. Memory B cells can be found in cord blood but in low amounts as compared to adult blood [55].

### Development of ILCs

ILCs are lymphocytes with an innate character that share transcriptional and effector functions with T cells [18]. Three types of ILCs can be distinguished: Group 1 (including NK cells), 2, and 3 ILCs [18]. Since lymphocytes and ILCs have the same progenitors, also the ILC progenitors first originate from the yolk sac and aorta [23–25] and migrate to the fetal liver [33, 34], where these cells can be found at 3–4 weeks of pregnancy [74]. Specific ILC progenitors and ILC subpopulations can be found in the fetal liver between weeks 6–12 of pregnancy [75, 76]. However, the ILC progenitors not only home to the fetal liver, but also to other tissues, such as the lung and intestine [75]. NK cells (group 1 ILCs) can already be found in the fetal liver at week 6–9 of pregnancy and in the fetal spleen at week 15 of pregnancy [76].

In human cord blood, the percentage of ILCs is higher when compared with adult blood, but still very low, i.e. below 1% [77](Table 1). The most well-known ILC, the NK cell is best characterized in cord blood. NK cell count in cord blood is higher when compared with adults [78, 79], with no significant difference in the percentage of NK cell subsets [79]. Cord blood NK cells have phenotypic features of immature NK cells [79] and show lower cytotoxic activity in vitro [79].

### Development of macrophages, dendritic cells, granulocytes, and monocytes

The yolk sac-derived macrophage progenitors migrate to the fetal tissues and develop into fetal macrophages, without developing into monocytes first [80–82]. They remain the major macrophage type that populates the adult brain [81, 83]. Fetal dendritic cells have been identified in the early human fetus, as of 9 weeks of gestation in the skin [84, 85]. The number of dendritic cells increases over gestation [84] and all subsets of dendritic cells (both lymphoid and myeloid) have been identified by 13 weeks of gestation [86].

Granulocytes and monocytes are derived from the EMPs from the yolk sac [87, 88]. The first monocytes are found from embryonic day 12 in the mouse and can be found in the fetal circulation until embryonic day 17 [87]. They colonize all fetal tissues, apart from the brain, where they differentiate into macrophages [30, 87]. These macrophages are thought to replace the fetal macrophages arising from the first yolk sac-derived primitive progenitors in most tissues, except for the brain, and remain in the tissues in adult life [87, 89–93]. A second wave of monocytes is derived from HSCs after embryonic day 17 in the mouse. This wave accounts for a minority of monocytes in fetal mice [87]. Granulocytes are limited in number in the first 4 months of gestation. After establishment of HSCs in the fetal bone marrow, the number of granulocytes in the fetal circulation increases about fourfold [88].

In human cord blood at birth, the granulocyte count and monocyte count are higher as compared with adult blood [54] (Table 1). However, these cells show phenotypical and functional differences with adult cells, such as differences in toll-like receptor expression and surface expression of adhesion molecules [94–96]. Cord blood granulocytes also showed decreased responses to chemotactic factors [97]. The monocyte subset distribution is similar in cord blood as compared with adult blood [98]. Cord blood monocytes showed similar expression of activation markers as compared with adult monocytes [98]. Furthermore, monocyte cytokine responses to various stimuli are found to be deficient or normal, depending on the stimulus and the cytokine [98–101]. In addition, the numbers of dendritic cells in cord blood are decreased as compared with adult blood [102–104]. Dendritic cells showed a higher degree of immaturity [102].

### The fetal immune response

It is apparent from the above-described fetal immune cell development that early in pregnancy fetal immune cells develop and circulate. It has long been thought that a fetal immune response did not exist and that fetal immune cells, although present, were not able to mount an immune response to external stimuli or foreign cells [105–107]. This was believed due to the absence of antigenic stimulation and the sterile environment of the developing fetus. This was also thought to be important for fetal development and acceptance since an adult-like immune response in the fetus would compromise fetal tolerance. However, various studies have now shown that fetal lymphocytes are not inert and perfectly able to respond to various stimuli, including to PHA [108] and allogeneic cells [44, 109]. Furthermore, a subset of fetal T cells consistently expressed CD69, a membrane-bound protein, which is upregulated within hours following stimulation of the T cell receptor [42]. Together, these data suggest that fetal T cells are not inert, but able to respond to various stimuli and that a subset of T cells is activated *in utero*. These could be autoreactive T cells, i.e. T cells responding to the fetus’s own cells, T cells responding to maternal cells [42], or T cells responding to bacterial products, since both maternal cells [13] and bacteria or bacterial products (see below) can cross the placenta into the fetus.

Activation of fetal T cells may be dangerous for fetal development, since activated T cells may damage fetal tissues. Therefore, a mechanism is in place to inhibit T cell activation in the fetus. As described above, Treg cells can be found in the fetal circulation. The amount of Treg cells in the fetal circulation is higher as compared to the amount of Treg cells in adults [13, 42, 44, 109]. The increased numbers of Treg cells in the fetal circulation are the result of a greater tendency of naïve fetal T cells to develop into Treg cells as compared with adult T cells [13, 45]. Upon stimulation, naïve fetal T cells rather develop into Treg cells than into effector T cells. This is a unique feature of fetal T cells. There are not only more Treg cells in the fetal circulation, but they also have an increased suppressor activity [45]. The importance of the presence of Treg cells in the fetal circulation was shown by Michaelsson et al., who showed that in vitro fetal T cells readily proliferate, i.e., become activated, when Treg cells were depleted from the culture. This indicates that Treg cells actively suppressed fetal T cells [42].

The finding of low numbers of memory T cells in cord blood [46, 110], fetal spleen [111] and after delivery in neonates is in line with the hypothesis that fetal T cells can respond to various stimuli, since memory T cells only develop after exposure to an antigen [112]. These fetal memory T cells have the same phenotypical markers as adult memory T cells, like CD45RO [46, 110]. They can also be activated and produce various cytokines upon activation [46]. However, the production of both Th2 cytokines, such as IL-4 and IL-13, and the Th1 cytokine IFNγ [46], indicates a more innate phenotype rather than an adaptive phenotype. In the fetal intestines, a larger number of memory T cells have been found as compared with fetal blood [47–49]. These memory T cells are suggested to play an important role in intestinal development [49]. The presence of these memory T cells suggests the presence of bacteria or their products in the fetal intestine (see Sect. 4).

The fact that fetal dendritic cells are also present in early development may suggest an early role for these cells as APC for the T cells. Although fetal dendritic cells migrate to lymph nodes and respond to toll-like receptor ligation similar to adult dendritic cells, they differ in their response to allo-antigens [113]. Rather than inducing an immune response toward the allo-antigen, fetal dendritic cells strongly promote Treg cell induction and inhibit T cell TNFα production, i.e. inhibit proinflammatory responses [113]. Thus, fetal dendritic cells are important for tolerance of maternal antigens.

Not only fetal T cells and dendritic cells are programmed toward tolerance of maternal antigens, also fetal monocytes seem to be programmed toward tolerance of maternal allo-antigens. IFNγ stimulation of fetal monocytes did not result in upregulation of the antigen presentation and co-stimulatory machinery in fetal monocytes as it does in adult monocytes [114]. This study also showed that stimulation of fetal monocytes with various cytokines (IL-6, IFNγ, IL-4) induced activation of a different transcriptional profile and different phosphorylation of signal transducers and activators of transcription as compared to similar activation of adult monocytes [114]. These data indicate that fetal monocytes are distinct from adult monocytes and that fetal monocytes support antimicrobial immune responses, and do not promote adaptive immune responses toward self or maternal allo-antigens.

## The influence of the maternal gut bacteria on the development of the fetal immune system

The factors promoting the development of the fetal immune system are not completely known. The presence of maternal cells in the fetus (microchimerism) is one of the factors thought to be responsible for promoting fetal immune system development [13]. Other factors may include substances from ingested amniotic fluid [115]. Also, maternal gut bacteria may be important for driving fetal immune maturation [14]. In this respect, it is interesting to note that the gut bacteria change during pregnancy. Such changes may be important for fetal immune maturation. Before we describe the potential effects of maternal gut bacteria on fetal immune maturation, we first discuss the changes in the maternal gut bacteria during pregnancy and the presence of bacteria or their products or metabolites in the fetus and placenta.

### Maternal gut bacteria changes during pregnancy

The adult microbiome in the human gut consists of more than 100 trillion microorganisms. These are bacteria, viruses, fungi, and archaea [116]. Most knowledge today has been acquired about the gut bacteria. The most abundant bacterial phyla in the human gut are Firmicutes, Bacteroidetes, Actinobacteria, Proteobacteria, Fusobacteria, and Verrucomicrobia, with Firmicutes and Bacteroidetes representing about 90% of all gut bacteria [117]. The gut bacteria are important for the host metabolism, but also for maturation of the immune system as well as for immune function during adult life. The gut bacteria do this, amongst others, by producing many metabolites, such as short chain fatty acids, that not only affect the gut bacteria, but that can also affect the immune system of the host [118].

Many host and environmental factors affect the gut bacteria, such as diet, drugs, age, and genetics [116]. In addition, pregnancy also affects the gut bacteria. Although various groups showed changes in the gut bacterial composition during pregnancy, different compositional changes were observed. The changes depend on gestational age [119, 120] and most changes were observed at the end of pregnancy. The bacterial load increases during the course of pregnancy [121], while the gut bacteria at the end of pregnancy showed a decreased α-diversity and an increased β-diversity [120, 122, 123]. At the end of pregnancy, various differences were observed at the phylum level compared to early pregnancy or non-pregnant women: an increased abundance of Actinobacteria and Proteobacteria was found by some groups [120, 124, 125], while another group found an increase in Bacteroidetes, with a decrease in Verrucomicrobia and Tenericutes [126]. One study found a decrease in the phylum TM7 [127]. Other studies found changes at the family or genera level. Some studies found an increase in *Bifidobacterium* species at the end of pregnancy [119–121, 123]). Also, a reduction in *Bacteroides*, with an increase in *Prevotella* [121, 123]) has been found at the end of pregnancy. *Faecalibacterium* were found to be increased [123] or decreased [120] in the second and third trimester of pregnancy. Genera like *Akkermansia*, *Ruminococcus*, *Dialister* and *Lachnospiraceae* decreased with gestational age [119, 124]. Even though most studies found changes in the maternal gut microbiome, one study did not find such changes [128].

In general, it seems that the maternal gut microbiome changes during pregnancy, however, there is no consensus on which exact changes do take place during pregnancy. Differences between various studies may be due to different methods of measuring the bacteria (different methods of DNA isolation, primers to different variable region of the 16 S rRNA are used, different taxonomic assignment), timing of stool samples (most changes take place in the third trimester). In addition, differences in study populations may affect the results since Xiao et al. found differences in pregnancy-induced changes in the gut bacteria between Western and non-Western populations [123]. A main factor affecting the maternal microbiome during pregnancy may be the diet [129, 130]. Various dietary components, such as high intake of carbohydrates, fat, or proteins have shown to affect Firmicutes, Actinobacteria, Proteobacteria, *Bacteroides*, or *Roseburia* in pregnancy (reviewed by Kunasegaran et al. [129]).

Since it is difficult to standardize the maternal diet during human pregnancy, it is also difficult to separate the effects of diet and pregnancy perse on the changes in the maternal gut bacteria during pregnancy in humans. Therefore, animal studies may help separating the effect of diet and pregnancy. Various animal studies have been performed, in which the maternal diet was similar before and during pregnancy and these studies also showed changes in the maternal gut bacterial composition during pregnancy, indicating that there is an effect of pregnancy perse on the maternal microbiome. Gohir et al. identified various genera that were different between pregnant and non-pregnant mice, including *Akkermansia*, *Bacteroides*, and *Bifidobacterium*, which were all increased during pregnancy in mice fed a standardized diet before and during pregnancy [131]. Also, our own study in pregnant mice showed that in mice fed a standardized diet before and during pregnancy, changes in the gut bacteria were found during pregnancy, especially at the end of pregnancy, with increased Firmicutes and decreased Bacteroidetes and changes in various species [132]. Not only in mice, but also in rats, fed a standardized diet before and during pregnancy, pregnancy-induced changes in the gut bacteria were found [133]. In addition, animal studies have also shown that different diets lead to a different maternal microbiome. For example, a high-fat diet in mice before and during pregnancy promoted shifts in maternal gut bacteria as compared with pregnant mice fed a control diet. A study by Gohir et al. found a decreased abundance of *Lachnospiraceae* and *Ruminococcaceae* in pregnant mice fed a high-fat diet compared to pregnant mice fed a control diet [131], while a study by Wekema et al. found decreased abundance of various bacterial genera in mice fed a high-fat diet during pregnancy, including *Bifidobacterium* and *Christensenella*, compared with pregnant mice fed a control diet [134].

The changes in gut bacteria during pregnancy raises the question whether these changes are a response to the numerous physiological changes that take place during pregnancy or whether they play an active role in driving these physiological changes in the mother during pregnancy or in supporting fetal development. It is likely not a simple one-way relationship; there may be a mutual interaction between physiological changes in pregnancy and changes in the gut bacteria during pregnancy. For instance, the important pregnancy hormones, progesterone and estrogen, can modulate the gut bacteria, but these hormones can also be modulated by the gut bacteria [135–137]. Furthermore, while the microbiome is important in developing and maintaining the immune system, the immune system itself affects the microbiota (reviewed by Zheng et al. [138]). It is likely that also during pregnancy a similar mutual interaction exists between the maternal immune response and the maternal gut bacteria. This is in line with our recent finding that the maternal gut bacteria are involved in adapting the maternal immune response to pregnancy [132]. Additionally, apart from influencing the maternal immune response, maternal gut bacteria may also play an important role in shaping the fetal immune response during pregnancy. This will be discussed in the following sections.

### Presence of bacteria or their products/metabolites in the placenta and fetus

The development of the fetal immune response *in utero* suggests that the fetus may not be sterile. However, there has been an ongoing debate whether bacteria are found in the placenta and the fetus. Various studies have shown the presence of bacterial DNA in the placenta [139–145] as well as in the fetus [146–148]. However, other studies could not find the presence of bacteria in the fetus or placenta and have suggested that the presence of bacteria in the placenta or fetus was due to contamination during labor or contamination by laboratory reagents [149–151]. Unfortunately, the above-mentioned studies did not culture the bacteria and therefore, solid evidence of the absence or presence of life bacteria in the placenta and fetus is lacking. Recent papers, which included various controls to rule out contamination, showed that life bacteria were present in the placenta, amniotic fluid as well as in various fetal organs in the second trimester of pregnancy, including in the lung and gut [14, 148, 152]. However, the setup of these studies was not sufficient to conclusively show that bacteria are present in the fetus, as indicated by a recent review [153]. It seems therefore unlikely that bacteria are present in fetal and placental tissue.

The maternal gut bacteria may still be able to affect the fetal immune response, potentially through bacterial products, such as bacterial DNA, LPS, or bacterial exosomes. These products can be found in the placenta, fetus, and meconium [140, 142, 146, 152, 154, 155]. Bacterial metabolites, such as short-chain fatty acids (SCFAs), are also able to affect immune responses and can be found in the fetus. In view of the changes in the maternal gut bacteria during pregnancy, especially changes in SCFA-producing bacteria, such as *Bifidobacterium*,* Prevotella*,* Rumminunococcus*, and *Lachnospiraceae* [156], it is likely that also the production of SCFA changes during pregnancy. A few studies have shown increased levels of SCFAs in the maternal gut or blood during pregnancy. The concentrations of fecal SCFA, as well as the concentration of SCFA in the cecum, were shown to be higher during pregnancy as compared to non-pregnant individuals [157, 158]. In mice and rats, acetate, propionate, and lactate were increased in the cecum or serum during pregnancy as compared with the non-pregnant state [158, 159]. Further research is needed to determine the maternal levels of SCFAs during pregnancy. This is important, since SCFA are able to pass the placenta [14, 160], and are able to affect the fetal immune response. Indeed, one study in humans found SCFAs in cord blood [160] and some studies in mice also found SCFAs in the fetus [161, 162].

Other bacterial metabolites, which are able to affect the immune response, such as secondary bile acids and tryptophan derivatives can also be detected in the fetus [161, 163]. Since bacterial genera known for their potential to transform primary bile acids to secondary bile acids, such as *Bacteroides* and *Bifidobacterium* [164], have been shown to change during pregnancy, it seems likely that also the production of secondary bile acids has changed during pregnancy. Unfortunately, there are no studies confirming this suggestion. Bacterial metabolites derived from the metabolism of tryptophan, such as indoles, can also be found in the fetus [161]. Various *Bacteroides* and *Bifidobacterium* species, which are increased in pregnancy, have been shown to metabolize tryptophan [165], suggesting the tryptophan metabolism has also changed in pregnancy. This suggestion remains to be investigated.

### The effect of bacterial products/metabolites on (fetal) immune cells?

One important study highlighting the impact of bacteria in the maternal gut on the fetal immune response is the study of Gomez de Aguero et al. [166]. This study demonstrated that maternal colonization of germ-free mice with E-coli altered the immune cell composition of the neonatal intestines 14 days after birth: group 3 ILCs and macrophages were increased in the offspring. Additionally, the intestines of the offspring of pregnant mice colonized with E-coli exhibited an enhanced response to microbial invasion after birth, while the intestinal immune response was less active to proinflammatory microbial products and microorganisms. This suggests that the immune system and the intestines were better prepared for extrauterine life after birth [166]. Since the germ-free mice were only colonized with the E-coli bacteria during pregnancy, this study suggests an effect of E-coli colonization on the fetal immune response. Unfortunately, these authors did not investigate changes in the fetal immune response itself.

Research regarding the effects of SCFAs on immune cells has predominantly focused on adult immune cells. It has been shown that SCFAs boost Th1 and Th17 responses and increase the production of IL-22 and IL-17 during immune responses [167]. Additionally, SCFAs play a role in the production and function of Tregs [168]. They also influence innate immune cells, such as dendritic cells and macrophages [169, 170]. Moreover, secondary bile acids and tryptophan metabolites, such as indoles, also significantly impact immune cells. Secondary bile acids affect innate immune responses [171], and adaptive immune responses [172]. Indoles, for instance, decrease pro-inflammatory cytokines, like IL-8, while promoting the production of anti-inflammatory IL-10 [173, 174]. They have also been shown to increase the number of regulatory T cells and decrease Th17 cells [175, 176]. However, future research is necessary to evaluate the effects of SCFAs, tryptophan metabolites and secondary bile acids on fetal immune cells.

There is limited knowledge about the effects of microbial products or metabolites on the fetal HSCs. However, a study on germ-free fish showed abnormal development of HSCs in germ-free fish, showing a slight reduction of HSCs in the AGM region, with greater reductions later in development [177]. This study also indicated that intestinal dysbiosis in the embryonic fish negatively impacted HSC development. These findings align with existing evidence regarding the influence of microbial products and metabolites on HSCs and immune cell development in the bone marrow in neonates and adults (reviewed by McCoy et al. [178]).

### Maternal gut dysbiosis and fetal immune response

If the maternal gut bacteria play a crucial role in the development of the fetal immune response, it can be hypothesized that maternal gut dysbiosis may be linked to abnormal development of the fetal immune response. Several clinical observations support this hypothesis. For instance, both obesity and atopy during pregnancy are associated with maternal gut dysbiosis [121, 179]. Offspring of obese and atopic mothers have an increased incidence of asthma and wheezing [180], suggesting disruptions in the development of fetal immune cells. Indeed, leukocyte subsets in the cord blood of neonates of obese and atopic women are different as compared to these subsets in healthy pregnant women: changes in the leukocyte subsets, such as Treg cells, in the cord blood of neonates from obese or atopic mothers were found [181–186].

Women with pregnancy complications, such as preeclampsia or gestational diabetes mellitus (GDM) have gut dysbiosis [187–189]. Their offspring have an increased risk of developing asthma, eczema, and allergies [190, 191], suggesting abnormal development of the immune response *in utero*. This notion is supported by studies indicating that cord blood from preeclamptic women showed significant changes in the immune cell composition, including altered NK cell subsets, decreased levels of Th2 cells, Treg cells and neutrophils [192–195]. Similarly, cord blood from women with GDM showed modifications in leukocyte subsets, such as changes in the numbers of CD3 + or CD4 + T cells or CD8 + T cells [196, 197] as well as increased markers of Th1 cells [198]. Moreover, maternal treatment with antibiotics, which induces gut dysbiosis [199], has been associated with increased risk of eczema, food allergy, or asthma in the offspring [200–202], suggesting that antibiotic treatment may impact the fetal immune system. This suggestion is corroborated by our recent research, which demonstrated that maternal antibiotic treatment in mice led to maternal gut dysbiosis and alterations in fetal monocyte subsets as well as activation of fetal monocytes [203].

Overall, the data suggest that maternal gut bacteria play a crucial role in the development of the fetal immune response. However, none of the existing studies have directly measured fetal immune cells or cord blood immune cells in conjunction with the bacteria in the maternal gut or with microbial products. This limitation makes it challenging to establish causal relationships between maternal gut dysbiosis and fetal immune responses at this time. Two studies linked specific maternal gut bacteria to allergies in the offspring. One study found that the presence of total aerobes and Enterococci in the maternal gut were associated to an increased risk of wheezing in the infants [204]. Another study showed that the presence of *Prevotella copri* in the maternal gut predicted food allergies in the offspring [205]. It remains to be determined whether these bacteria are connected to specific changes in the fetal immune response.

### Maternal treatment with pre- or probiotics and the effects on the fetal immune response

Although, to date, there is limited evidence linking specific maternal gut bacteria or bacterial products or metabolites to the development of the fetal immune response, there is circumstantial evidence supporting a role of the maternal gut microbiome in this process. For instance, as discussed above, maternal dysbiosis has been associated with an increased incidence of allergies in the offspring. Additional circumstantial evidence arises from studies where the maternal gut microbiome is modified by supplementation with pre- or probiotics during pregnancy followed by assessments of the fetal immune response or allergy development in the offspring. Various studies have shown that treating pregnant women or rodents with prebiotics, such as galactooligosaccharides (GOS) or fructooligosaccharides (FOS), reduces the incidence of allergies or allergic symptoms in their offspring [206–209]. GOS and FOS are fermented by gut bacteria, such as *Bifidobacterium*, resulting in the production of SCFA, such as acetate [210]. Supporting these findings, two mouse studies from the same research group, observed that treatment of pregnant mice with GOS and inulin increased the numbers of regulatory T and B cells in the fetus [162] and offered protection against wheat allergy development [211]. Additionally, a recent study by Hu et al. found that the SCFA, acetate, administration during pregnancy improved thymic and Treg cell development in the fetus [194]. This suggests that one potential mechanism by which GOS treatment during pregnancy affects the fetal immune response may involve increasing acetate levels. Whether FOS and GOS treatment during human pregnancy also impacts fetal immune cells remains unclear, since in healthy pregnant women, supplementation with a mixture of FOS and GOS during pregnancy did not affect T cell subpopulations in cord blood [212].

Not only prebiotic supplementation, but also supplementation of probiotics, such as *Lactobacillus* or *Bifidobacterium*, suppressed allergic incidence or symptoms in the offspring in both mice and humans [213, 214]. Whether the effect of probiotics on allergy incidence or symptoms is induced by changes in the fetal immune response remains to be established. Only one study evaluated the effect of *Lactobacillus* treatment on cord blood immune cells and failed to find an effect [215].

## Final considerations

In summary, it is clear from the above that the fetal immune system differs from the adult immune system. Rather than the previous belief that the fetal immune system is inert, the fetal immune system develops in an environment in which self, maternal, and environmental antigens are present. To be able to survive, the fetal immune system must be uniquely equipped to tolerate these antigens. The fetal immune response is thus geared toward tolerance against maternal and allo-antigens and does not induce a proinflammatory response toward these antigens. At the same time the fetal immune response, when needed, can respond to infections and microbes. This is accomplished by a well-equipped innate immune response, consisting of, amongst others, fetal monocytes, CD8 + T cells and memory T cells, having an innate-like character, ILCs and γδ T cells.

This unique fetal immune response is crucial for fetal tolerance and development and therefore should be carefully orchestrated. Several factors are believed to contribute to this process, such as microchimerism, swallowing of substances from the amniotic fluid, and maternal bacteria. In this review, we focused on the role of maternal bacteria in shaping the fetal immune response. While the debate on the presence of bacteria in the placenta and fetus remains unresolved, it seems likely that the fetus does not contain living bacteria. Although direct evidence linking specific bacterial strains to fetal immune cell development is lacking, substantial circumstantial evidence suggests that maternal gut bacteria significantly influence the fetal immune response. For instance, dysbiosis of the maternal gut is associated with an increased risk of allergies in the offspring and alterations in immune cells in the cord blood. Moreover, treatment of pregnant individuals and mice with pre- or probiotics has been shown to reduce the incidence of allergies in the offspring. In mouse studies, these treatments correlate with changes in fetal immune responses, however, whether this is also true in human remains to be established. Overall, these findings highlight a potential role for maternal bacteria in the development of the fetal immune response, warranting further investigation.

We hypothesize that the changes in the maternal gut bacteria during pregnancy are important for development of the fetal immune system. It is interesting to note that alterations in the maternal gut bacteria appear to be influenced by gestational age, with the most significant changes occurring toward the end of pregnancy. In contrast, early pregnancy shows relatively few changes in the gut bacteria compared with non-pregnant women. This may suggest that the gestational age-dependent changes in the gut microbiome correlate with the developmental stages of the fetal immune system and that the initial stages of hematopoiesis may require a different maternal microbiome compared to the later stages of immune cell development.

Future studies should focus on evaluating the effect of bacterial products or metabolites on fetal immune cells precursors and fetal immune cells. In vitro experiments are necessary to assess the direct effects of bacterial products and metabolites on immune cell precursors and immune cells. In addition, germ-free animal models provide an excellent opportunity to study the in vivo role of maternal bacteria in shaping fetal immune response. Gnotobiotic models can shed more light on the contributions of specific bacterial species to the development of the fetal immune response. It is essential to recognize that it is not the bacteria themselves but rather their products and metabolites that affect the fetal immune response. This suggests that there may be a redundancy in the microbiome; different bacterial species of genera may produce similar metabolites that have comparable effects on the fetal immune system. Consequently, studies should also focus on metabolomics and the presence or absence of specific bacterial clusters producing certain products or metabolites. This approach will enhance our understanding of how the gut microbiota impact the fetal immune response.

Another key area for future research is the evaluation of the causal relationship between the fetal immune response and the adult immune response later in the offspring’s life. There is some evidence that an abnormal cord blood immune response is linked to the development of atopy in children [216, 217]. However, it remains unclear whether aberrant cord blood immune responses translate to aberrant child or adult immune responses and whether this ultimately translates into immune-mediated disease in the offspring. To address these questions, future research should investigate the neonatal, child, and adult immune response of fetuses with aberrant cord blood immune responses. Both animal studies and longitudinal human studies are essential to track the outcomes of the offspring with such immune anomalies over time.

The role of the maternal gut microbiota in the development of the fetal immune response opens new opportunities for optimizing the fetal immune response during pregnancy. Evidence linking pregnancy complications, such as preeclampsia, to gut dysbiosis and immune-mediated diseases in offspring suggests that improving the maternal gut microbiota could be a viable treatment option. Methods to improve the maternal gut microbiota are dietary changes and treatment with pre- or probiotics. Such treatments are cost-effective and safe during pregnancy [218] and have already been used during pregnancy. We hypothesize that these interventions improve the microbiota of pregnant women with dysbiosis, thereby improving the fetal immune response and reducing the risk of developing immune-mediated diseases in their offspring. While it has been demonstrated that pre- or probiotic treatment can decrease the incidence of allergies in the offspring, whether these treatments also affect the fetal immune response remains to be established. Therefore, future research should investigate the impact of pre- or probiotics on fetal immune cell development.

## Data Availability

Data sharing is not applicable to this article as no datasets were generated or analysed during the current study.
